# Profiling exercise intensity during the exergame Hollywood Workout on XBOX 360 Kinect®

**DOI:** 10.7717/peerj.5574

**Published:** 2018-08-30

**Authors:** Ricardo B. Viana, Rodrigo L. Vancini, Carlos A. Vieira, Paulo Gentil, Mário H. Campos, Marilia S. Andrade, Claudio Andre B. de Lira

**Affiliations:** 1 Faculdade de Educação Física e Dança, Universidade Federal de Goiás, Goiânia,Goiás, Brazil; 2 Centro de Educação Física e Desportos, Universidade Federal do Espírito Santo, Vitória, Espírito Santo, Brazil; 3 Departamento de Fisiologia, Universidade Federal de São Paulo, São Paulo, São Paulo, Brazil

**Keywords:** Physiological responses, Oxygen uptake, Heart rate

## Abstract

**Background:**

Despite the increasing popularity of exergame practice and its promising benefits in counteracting physical inactivity, limited research has been performed to document the physiological responses during an exergame session. This study aims (i) to investigate the responses of heart rate (HR) and oxygen uptake (${\dot{\rm V}}{{\rm{O}}_{\rm{2}}}$) during an exergame session and to compare with HR and ${\dot{\rm V}}{{\rm{O}}_{\rm{2}}}$ measured during joystick session and (ii) to compare HR and ${\dot{\rm V}}{{\rm{O}}_{\rm{2}}}$ obtained during exergame and joystick session with those HR and ${\dot{\rm V}}{{\rm{O}}_{\rm{2}}}$ associated with first and second ventilatory thresholds (VT_1_ and VT_2_, respectively) obtained during a maximal graded exercise test.

**Methods:**

A total of 39 participants performed a maximal graded exercise test to determine maximal oxygen uptake (${\dot{\rm V}}{{\rm{O}}_{\rm{2}}}\max $), VT_1_, and VT_2_. On separate days, participants performed an exergame and traditional sedentary game (with a joystick) sessions. The time that participants remained with HR and ${\dot{\rm V}}{{\rm{O}}_{\rm{2}}}$ below the VT_1_, between the VT_1_ and VT_2_ and above the VT_2_ were calculated to determine exercise intensity.

**Results:**

Heart rate and ${\dot{\rm V}}{{\rm{O}}_{\rm{2}}}$ were below VT_1_ during 1,503 ± 292 s (86.1 ± 16.7%) and 1,610 ± 215 s (92.2 ± 12.3%), respectively. There was an increase in HR and ${{\dot {\rm V}}}{{\rm{O}}_{\rm{2}}}$ as a function of exergame phases, since HR mean values in the ‘warm-up’ period (119 ± 13 bpm) were lower than the ‘main phase’ (136 ± 15 bpm) and ‘cool-down’ periods (143 ± 15 bpm) (*p* < 0.001). Regarding ${\dot {\rm V}}{{\rm{O}}_{\rm{2}}}$ values, the ‘warm-up’ (25.7 ± 2.9 mL.kg^−1^.min^−1^) were similar to the ‘main phase’ (25.1 ± 2.8 mL.kg^−1^.min^−1^) (*p* > 0.05) and lower than the ‘cool-down’ (28.0 ± 4.8 mL.kg^−1^.min^−1^) (*p* < 0.001). For all times of the joystick session, average HR and ${\dot {\rm V}}{{\rm{O}}_{\rm{2}}}$ were below the VT_1_ levels.

**Conclusion:**

Exergames can be classified as light to moderate exercise. Thus, exergames could be an interesting alternative to traditional forms of exercise.

## Introduction

Physical inactivity is associated with an increase in the risk of a variety of chronic diseases (such as diabetes mellitus and arterial hypertension) and consequently, premature deaths (Ding et al., 2016). Although well documented, a large part of the population remains sedentary (Fox, 2012). In this context, adults who are not engaged in traditional exercise methods with a minimum intensity corresponding to 55% of maximal heart rate (HRmax) require a strategy to achieve and maintain sufficient physical activity for health benefits (Garber et al., 2011).

Exergames, or active videogames, may motivate some adults to engage in physical activity and may be an attractive and fun alternative of physical activity for increasing the motivation of people to engage in an exercise programme (Street, Lacey & Langdon, 2017).

Exergames can be defined as electronic games that allow players to physically interact with images on screen (Foley & Maddison, 2010). Traditionally, exergames are characterized as physical activities including games that simulate walking, running, stair climbing, cycling, rowing, and swimming (Graves et al., 2010; Wu, Wu & Chu, 2015). Furthermore, there are games that require mixed activity that simulate jumping, throwing, and kicking (Bird et al., 2015).

Most studies involving exergames investigated the acute and chronic effects on caloric expenditure (Barkman et al., 2016), motor aspects (Collado-Mateo et al., 2017), and cognitive function (Garcia et al., 2016; Stanmore et al., 2017) in different healthy and clinical populations (Dos Santos Mendes et al., 2012; Collado-Mateo et al., 2017). However, less is known about the physiological responses during an exergame session, especially regarding oxygen uptake (${\dot{\rm V}}{{\rm{O}}_{\rm{2}}}$) and heart rate (HR). These physiological variables are a common form for gathering information about exercise intensity. Moreover, these physiological variables are useful tools to monitor responses and adaptations to exercise. In this regard, it has previously been shown that an increase in one metabolic equivalent in exercise capacity decreases mortality rate by 12% (Myers et al., 2002; Kodama et al., 2009).

Neves et al. (2015) investigated the acute cardiovascular responses during a session of the game *Zumba Fitness Core*® performed with XBOX 360 and observed a significant increase of HR immediately after the session. Graves et al. (2010) compared the physiological cost among adolescents, young, and older adults during a game with a joystick, *Wii® Fit* activities (yoga, muscle conditioning, balance, and aerobics), and brisk treadmill walking and jogging and found that energy expenditure and HR of *Wii® Fit* activities were greater than joystick games, but lower than treadmill exercise and that the *Wii®* elicited moderate intensity activity. Rodrigues et al. (2015) evaluated the acute metabolic and cardiovascular responses of healthy men during exergames with the *Nintendo Wii®* (obstacle course, hula hoop, free run, soccer heading, penguin slide, and table tilt) and found that the *Wii®* exercises are considered to be of light and moderate intensity. Wu, Wu & Chu (2015) examined and compared the energy expenditure and intensity of XBOX 360 Kinect® exergames (boxing, soccer, track and field, ping pong, beach volleyball, and bowling) in healthy young adults and observed that boxing and soccer exergames provided the greater exercise intensity. It is important to note that none of these studies determined exercise intensity based on metabolic thresholds obtained in a continuous laboratory treadmill test, such as maximal graded exercise testing (GXT) (De Lira et al., 2013).

Thus, despite the increasing popularity of exergame practice (Sween et al., 2014) and its promising benefits in counteracting physical inactivity, limited research has been performed to document the physiological responses during an exergame session, which makes it difficult to characterize the physiological responses and brings uncertainty regarding its potential benefits in increasing physical fitness. Therefore, the aims of this study were to investigate physiological parameters of young men, to describe HR and ${\dot{\rm V}}{{\rm{O}}_{\rm{2}}}$ responses during the exergame *Hollywood Workout on* *XBOX 360 Kinect* session, and to compare with responses in a joystick game. Also, the study compared HR and ${\dot{\rm V}}{{\rm{O}}_{\rm{2}}}$ responses during exergame with those HR and ${\dot{\rm V}}{{\rm{O}}_{\rm{2}}}$ associated with first and second ventilatory thresholds (VT1 and VT2, respectively) attained during a maximal graded exercise test.

The exergame *Hollywood Workout on XBOX 360 Kinect* was chosen with the intention of maintaining ‘ecological validity,’ as this game simulates some exercises, such as, push-ups, skater lunges, and bicycle crunches that are performed in exercise facilities.

## Materials and Methods

### Participants

A total of 39 young men were recruited among students from the Faculty of Physical Education and Dance of the Federal University of Goiás (Brazil). Participants (25.9 ± 4.9 years, 1.79 ± 0.08 m, 79.2 ± 13.8 kg, 24.7 ± 3.4 kg.m^−2^) were recruited through social media and direct contact. Participants were physically active, asymptomatic and non-users of exergames. All participants were informed of the intent, experimental procedures, benefits, and risks of the study and informed consent was obtained from all individual participants included in the study. All experimental procedures were approved by the University Human Research Ethics Committee (no 1.459.010) and conformed to the principles outlined in the Declaration of Helsinki.

### Baseline examinations

Before the beginning of the experiment, each participant came to the laboratory for anamnesis through the *Physical Activity Readiness Questionnaire* (PAR-Q). The inclusion criteria were to respond ‘no’ on all the PAR-Q questions. No participants were excluded.

### Study design

Each participant reported to the laboratory on three separate days. The first day involved baseline examinations and GXT, and the other 2 days consisted of two randomly ordered sessions (exergame and joystick). The HR and ${\dot{\rm V}}{{\rm{O}}_{\rm{2}}}$ corresponding to VT_1_ and VT_2_ obtained in GXT were used as analysis parameters for the HR and ${\dot{\rm V}}{{\rm{O}}_{\rm{2}}}$ recorded during the exergame and joystick sessions. All sessions were started at the same time of day with at least 48 h separating the beginning of each visit. Participants were instructed to eat a standardized meal, not to participate in any strenuous exercise, and not to consume any stimulant or alcohol in the 24 h preceding all testing sessions. The temperature and relative humidity in the testing laboratory ranged from 21 to 23 °C and 55% to 65%, respectively, for all trials.

### Experimental procedures

#### Maximal graded exercise testing

**Figure 1:**
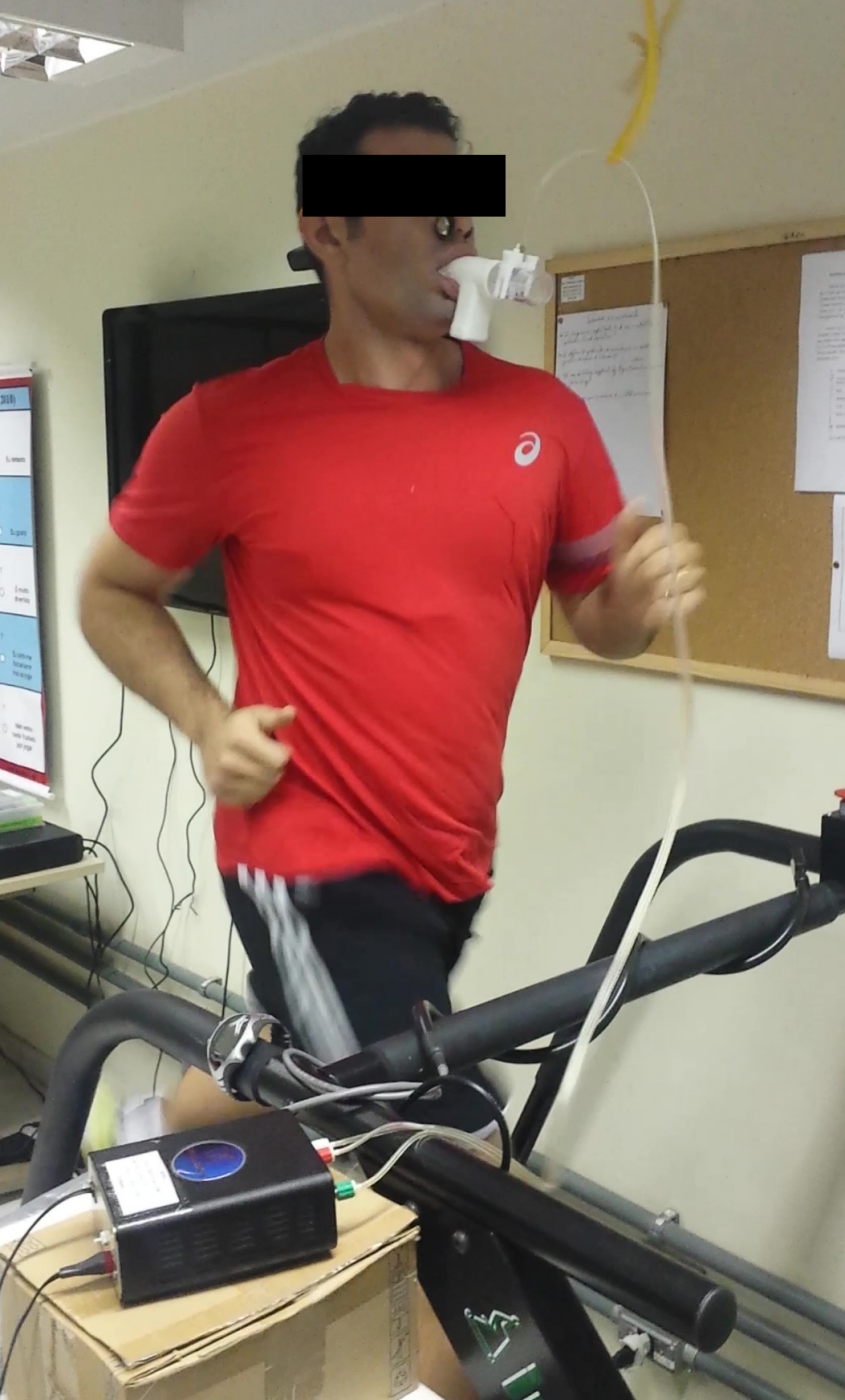
Maximal graded exercise testing session. Photo credit: Ricardo B. Viana.

Graded exercise testing was administrated to determine VT_1_, VT_2_, and maximal oxygen uptake (${\dot{\rm V}}{{\rm{O}}_{\rm{2}}}\max $), as well as their associated running velocities, HR and ${\dot{\rm V}}{{\rm{O}}_{\rm{2}}}$. Prior to performing the GXT, participants were given a standardized set of instructions explaining the test. On completion of these preliminary procedures, each participant underwent an incremental maximal exercise test on a motorized treadmill (ATL, Inbramed, Porto Alegre, Brazil) with 0% slope. The schedule of this test consisted of a 5-min warm-up period at seven km.h^−1^, and then the initial speed was progressively increased by one km.h^−1^ every minute until exhaustion (De Lira et al., 2013). During the exercise testing, participants were verbally encouraged to exercise for as long as possible. Respiratory gas samples were measured continuously using a metabolic system (VO2000; MedGraphics, Saint Paul, USA). Prior to testing, the metabolic system was calibrated according to the manufacturer’s instructions. HR was recorded using a HR-monitor (RS800CX; Polar Electro, Espoo, Kempele, Finland). Figure 1 shows a participant during the GXT.

The following data (averaged over 10 s) were obtained: ${\dot{\rm V}}{{\rm{O}}_{\rm{2}}}$ (mL.kg^−1^.min^−1^) at standard temperature (0 °C) and barometric pressure at sea level, carbon dioxide production (${\dot{\rm V}{\rm C}}{{\rm{O}}_{\rm{2}}}$) (mL.kg^−1^.min^−1^) at standard temperature (0 °C) and barometric pressure at sea level, respiratory exchange ratio (RER), minute ventilation (${{\dot{\rm V}}_E}$) (L.min^−1^) at body temperature and saturation pressure, respiratory rate (breaths per minute (bpm)), ventilatory equivalents for O_2_ and CO_2_ (${{\dot{\rm V}}_E}$/${\dot{\rm V}}{{\rm{O}}_{\rm{2}}}$ and ${{\dot{\rm V}}_E}$/${\dot{\rm V}{\rm C}}{{\rm{O}}_{\rm{2}}}$, respectively), expired fractions of O_2_ and CO_2_ (%) and HR (beats per minute (bpm)). Peak treadmill speed was defined as the last achieved running speed sustained for at least 30 s. ${\dot{\rm V}}{{\rm{O}}_{\rm{2}}}\max $ was defined as the highest 10-s averaged ${\dot{\rm V}}{{\rm{O}}_{\rm{2}}}$ value with inclusion criteria consistent with conventional guidelines for ${\dot{\rm V}}{{\rm{O}}_{\rm{2}}}\max $ (e.g. an inability to sustain the workload, relative HR > 95% predicted for their age, RER at maximal exercise ≥ 1.1, and ${\dot{\rm V}}{{\rm{O}}_{\rm{2}}}$ plateau [the point at which ${\dot{\rm V}}{{\rm{O}}_{\rm{2}}}$ increases less than 150 mL.min^−1^ for a given increase in workload]) (Howley, 2007). VT_1_ and VT_2_ were assessed using established criteria (Wasserman et al., 2005). Briefly, VT_1_ corresponds to the break point in the plot of ${\dot{\rm V}{\rm C}}{{\rm{O}}_{\rm{2}}}$ as a function of ${\dot{\rm V}}{{\rm{O}}_{\rm{2}}}$. At that point, ${{\dot{\rm V}}_E}$/${\dot{\rm V}}{{\rm{O}}_{\rm{2}}}$ increases without an increase in ${{\dot{\rm V}}_E}$/${\dot{\rm V}{\rm C}}{{\rm{O}}_{\rm{2}}}$. VT_2_ was located between VT_1_ and ${\dot{\rm V}}{{\rm{O}}_{\rm{2}}}\max $, when ${{\dot{\rm V}}_E}$/${\dot{\rm V}{\rm C}}{{\rm{O}}_{\rm{2}}}$ starts to increase and ${{\dot{\rm V}}_E}$/${\dot{\rm V}}{{\rm{O}}_{\rm{2}}}$ continues to increase. VT_1_ and VT_2_ were determined independently by two experienced investigators. If agreement between the investigators was not achieved, VT_1_ and VT_2_ were determined by consensus. To determine the ${\dot{\rm V}}{{\rm{O}}_{\rm{2}}}$ and ${{\dot{\rm V}}_E}$ at VT_1_ and VT_2_, the average of the last 10 s of each corresponding level was used. In practical terms, ventilatory thresholds represent points that can be used to classify the intensity of aerobic exercise.

#### Exergame session

The exergame session was conducted in a room (10 × 6 m) and was accompanied by a certified trainer that was experienced in exergames. For the purposes of the present study, the exergame *Hollywood Workout* (Majesco Entertainment, Edison, Hazlet, NJ, USA) was used with the intention of maintaining ‘ecological validity,’ as this game simulates some exercises usually performed in exercise facilities. Indeed, this exergame has several pre-established training protocols consisting of standardized exercises. Due to the feasibility of performing the exercises by coupling the participant to the metabolic system, the training protocol used in the present study was the *Sports Athlete*.

The *Sports Athlete* protocol consists of three phases: warm-up, main, and cool-down, with a total exercise time of 19 min and 28 s and total time in transitions between exercises of 8 min and 32 s, totalling a session of 28 min. However, total session time could vary according to the ability of the participant in the transitions between exercises. At the end of the *Sports Athlete* protocol, a numerical total score was provided. Table 1 provides the exercises used in *Sports Athlete* protocol.

**Table 1:** Sports athlete protocol of the exergame *Hollywood Workout*.

Phases	Exercises	Sets	Time (seconds)	Repetitions*
Warm-up	Jog	1	71	25
	Trunk crosses	1	62	20
	Jumping jacks	1	68	40
	Ice skaters	1	59	12
Main	Push-ups	1	59	12
	Skater lunges	1	69	16
	Bicycle crunches	1	74	25
	Push-ups	1	73	15
	Skater lunges	1	82	24
	Bicycle crunches	1	74	25
	Push-ups	1	74	15
	Skater lunges	1	81	24
	Bicycle crunches	1	74	25
Cool-down	Punches	1	61	25
	Jump rope	1	56	40
	Side shuffles	1	68	20
	Mountain climbers	1	63	25

**Note:**

* Repetitions proposed by the exergame. However, the participant was instructed to perform as many repetitions as possible.

**Figure 2:**
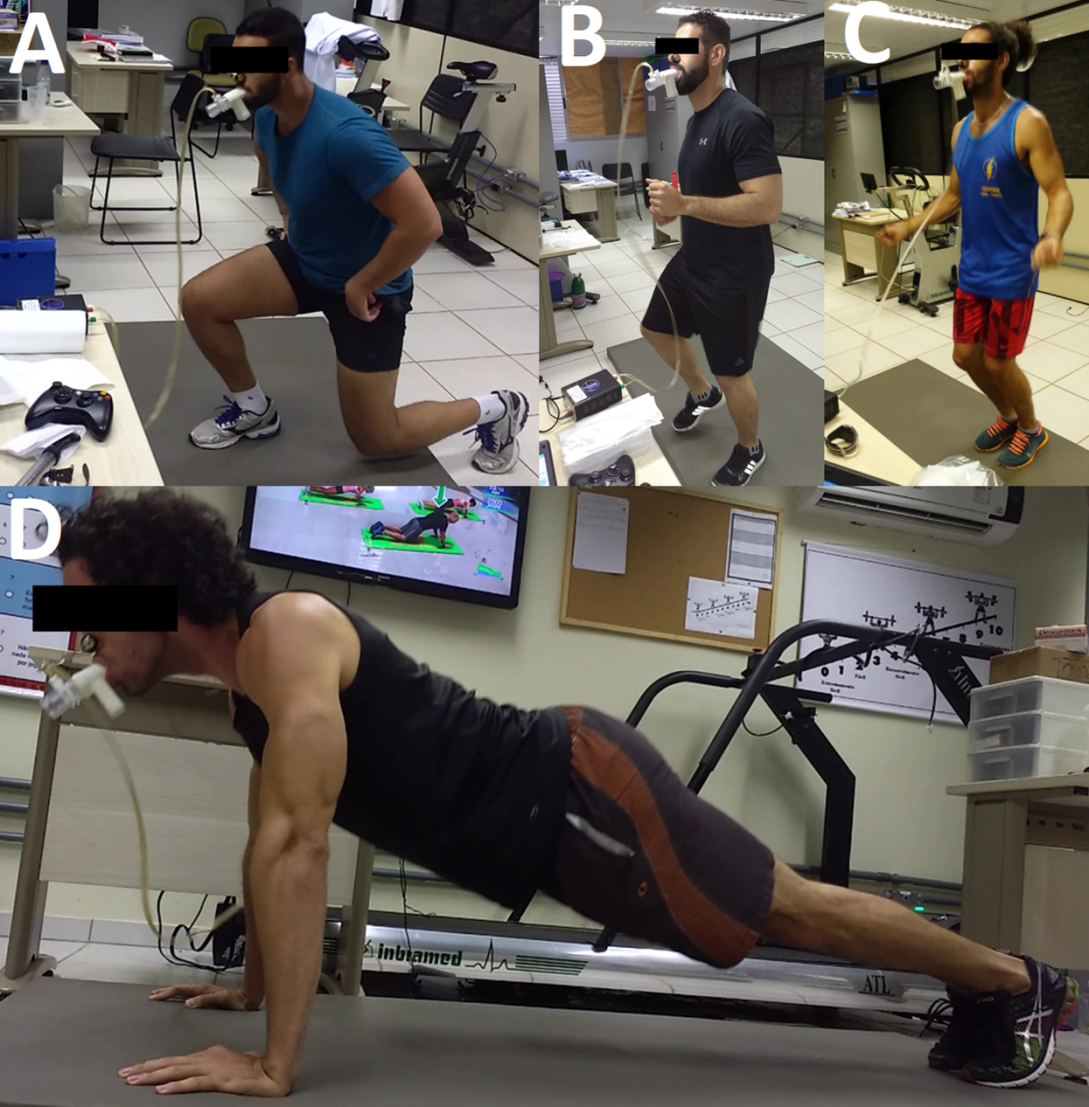
Some exercises of the Sports Athlete protocol performed by the participants during the exergame session. (A) Skater lunges; (B) jog; (C) jump rope; (D) push-ups. Photo credit: Ricardo B. Viana.

Heart rate and ${\dot{\rm V}}{{\rm{O}}_{\rm{2}}}$ were recorded before and during (including rest periods between sets) the *Sports Athlete* protocol. HR (5 s average HR value) and ${\dot{\rm V}}{{\rm{O}}_{\rm{2}}}$ (10 s average ${\dot{\rm V}}{{\rm{O}}_{\rm{2}}}$ value) registers were monitored by a HR-monitor (RS800CX; Polar Electronics, Finland) and metabolic system (VO2000; MedGraphics, USA), respectively. HR and ${\dot{\rm V}}{{\rm{O}}_{\rm{2}}}$ recorded during the *Sports Athlete* protocol were compared with HR and ${\dot{\rm V}}{{\rm{O}}_{\rm{2}}}$ corresponding to VT_1_ and VT_2_ obtained in GXT Fig. 2 shows participants performing the *Sports Athlete* protocol.

#### Joystick session

**Figure 3:**
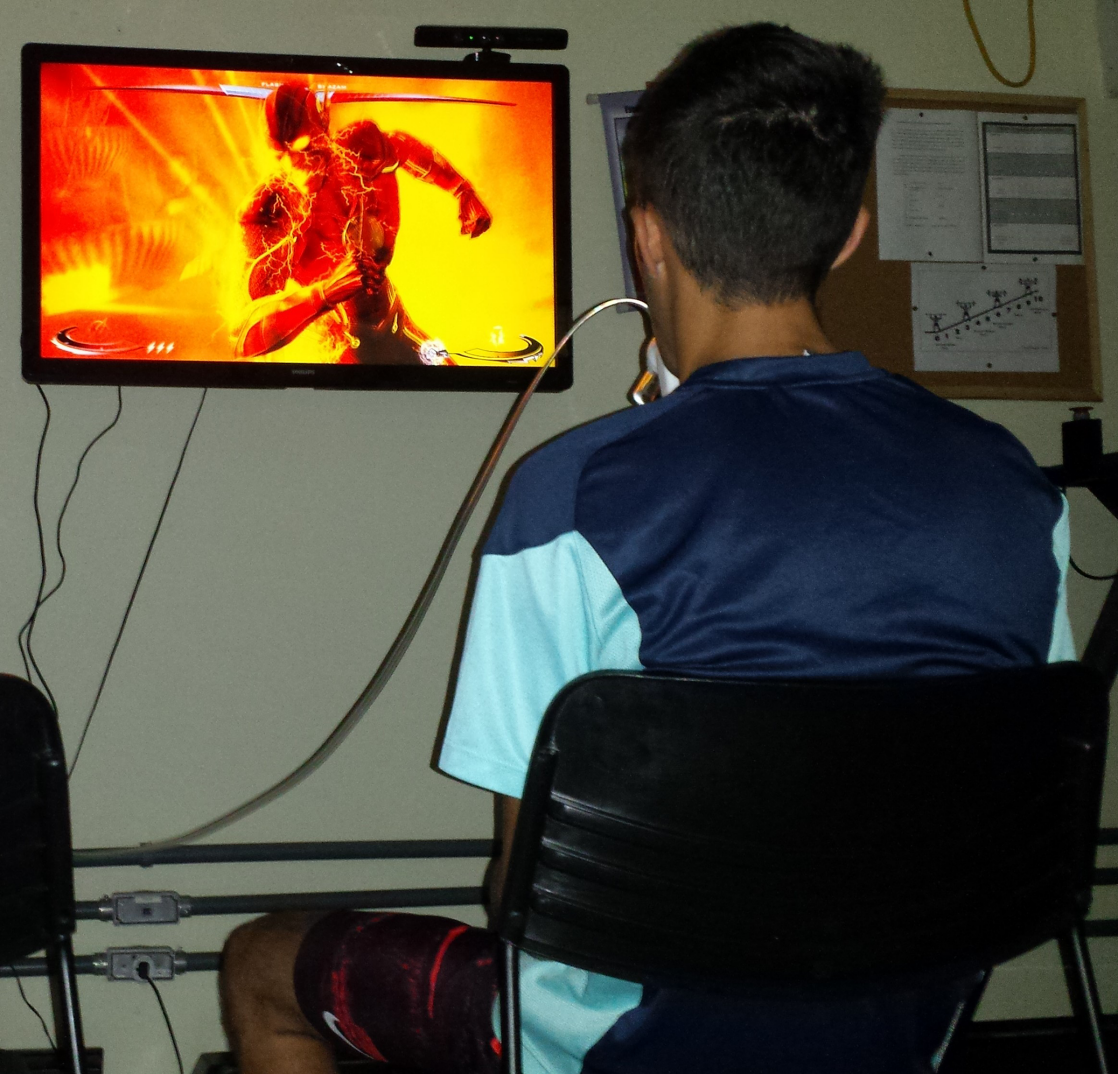
Joystick session. Photo credit: Ricardo B. Viana.

The participants also underwent a 25-min joystick game session (traditional sedentary video gaming) (Fig. 3). To reduce the effect of playing ability among participants was chosen the joystick-game *Injustice: Gods among us, ultimate edition*. This game is easy to play and allow the participants restart the fight easily and faster. We tried other joystick-games (fight games) but the participants dispended a lot of time between one fight and other. Indeed, we did not find a joystick-game similar with the exergame *Hollywood Workout*, since it is an exergame which simulates traditional physical exercises. HR and ${\dot{\rm V}}{{\rm{O}}_{\rm{2}}}$ values were recorded during all time sessions and compared with the HR and ${\dot{\rm V}}{{\rm{O}}_{\rm{2}}}$ corresponding to VT_1_ and VT_2_ obtained in GXT.

### Statistical analysis

Age, the highest HR and percent of maximal heart rate (HRmax), and average ${\dot{\rm V}}{{\rm{O}}_{\rm{2}}}$ in the exergame session presented a normal distribution (*p* > 0.05) according to the Shapiro–Wilk tests. All other variables in the exergame and joystick sessions presented a non-normal distribution (*p* < 0.05). Differences between the exergame and joystick sessions were analysed by the paired Student *t*-test (data with normal distribution) and Wilcoxon test (data with non-normal distribution). A one way repeated-measures analysis of variance was used to compare the differences between variables with normal distribution assessed in the ‘warm-up,’ ‘main phase,’ and ‘cool-down’ periods of the exergame session. When necessary, post hoc testing was performed by multiple comparisons using the Bonferroni procedure for confidence interval correction. The Friedman test was used to compare the differences between variables with non-normal distribution assessed in the ‘warm-up,’ ‘main phase,’ and ‘cool-down’ periods of the exergame session. The measures of the effect size for bilateral differences were calculated by dividing the mean difference by the standard deviation of the pre-training measurement. The magnitude of the effect sizes was judged according to the following criteria: *d = 0.2* considered a ‘small’ effect size; *0.5* represented a ‘medium’ effect size; and *0.8* a ‘large’ effect size (Cohen, 1988). All statistical analyses were performed with the Statistical Package for the Social Sciences version 20.0. The significance level was set *p* < 0.05. Data are shown as the means ± standard deviations.

## Results

### Maximal graded exercise testing

Data from the GXT are shown in Table 2. VT_1_ and VT_2_ were detected in all cases.

**Table 2:** Physiological variables obtained by maximal graded exercise testing.

GXT (*n* = 39)	${\dot{\rm V}}{{\rm{O}}_{\rm{2}}}$ (mL.kg^−1^.min^−1^)	$\% {\dot{\rm V}} {{\rm{O}}_{\rm{2}}}\max $	HR (bpm)	% Attained HRmax	Speed (km.h^−1^)
VT_1_	38.1 ± 5.9	60.9 ± 6.3	156 ± 12	82.0 ± 4.9	10.6 ± 1.2
VT_2_	48.7 ± 6.3	77.7 ± 5.7	172 ± 10	90.2 ± 3.7	13.2 ± 1.4
Maximal exercise	62.7 ± 7.4	N/A	191 ± 11	N/A	16.1 ± 1.7

**Notes:**

Data are presented as the mean ± standard deviation.

GXT, maximal graded exercise test; ${\dot{\rm V}}{{\rm{O}}_{\rm{2}}}$, oxygen uptake; ${\dot{\rm V}}{{\rm{O}}_{\rm{2}}}\max $, maximal oxygen uptake; HR, heart rate; HRmax, maximal heart rate; VT_1_, first ventilatory threshold; VT_2_, second ventilatory threshold; bpm, beats per minute; N/A, not applicable.

### Exergame session

Heart rate behaviour during the *Sports Athlete* protocol is shown in Table 3. The highest HR and percentage of HRmax attained in both the ‘main phase’ and ‘cool-down’ of the exergame session were about 11 ± 10%, higher than those attained in the ‘warm-up’ (*p* < 0.001 for both, effect size = 1.17–1.30 [large] for all comparisons). The average HR and percentage of HRmax attained in the ‘cool-down’ was 20.4 ± 11.8% and 5.2 ± 7.1%, higher than those attained in the ‘warm-up’ (*p* < 0.001, effect size = 1.71 and 1.76 [large], respectively) and ‘main phase’ (*p* < 0.001, effect size = 0.47 [small] and 0.51 [medium], respectively), respectively. The average HR and percentage of HRmax attained in the ‘main phase’ was about 14.6 ± 9.7%, higher than that attained in the ‘warm-up’ (*p* < 0.001, effect size = 1.21 and 1.33 [large], respectively). During the ‘warm-up,’ the average time spent at a HR below the VT_1_, between the VT_1_ and VT_2_, and above the VT_2_ was 289 ± 27 s, 10 ± 25 s, and 1 ± 4 s, respectively. In the ‘main phase,’ the average time spent at a HR below the VT_1_, between the VT_1_ and VT_2_, and above the VT_2_ was 947 ± 197 s, 119 ± 144 s, and 34 ± 87 s, respectively. During the ‘cool-down,’ the average time spent at a HR below the VT_1_, between the VT_1_ and VT_2_, and above the VT_2_ was 271 ± 83 s, 63 ± 66 s, and 14 ± 41 s.

**Table 3:** Heart rate, oxygen uptake and time (expressed as relative percentages) in each metabolic zone.

	Warm-up	Main phase	Cool-down	*p*
**HR**				
Highest HR (bpm)	149 ± 12	165 ± 15*	164 ± 13*	<0.001
% Attained HRmax	78.5 ± 6.3	86.5 ± 6.4*	86.4 ± 7.2*	<0.001
Average HR (bpm)	119 ± 13	136 ± 15^#^	143 ± 15*^#^	<0.001
% Attained HRmax	62.6 ± 6.7	71.4 ± 6.5^#^	74.9 ± 7.3*^#^	<0.001
Time below to HR at VT_1_ (%)	96.5 ± 9.0	86.1 ± 17.9*	78.4 ± 24.1*	<0.001
Time between to HR at VT_1_–VT_2_ (%)	3.3 ± 8.0	10.8 ± 13.1*	18.3 ± 19.2*	<0.001
Time above to HR at VT_2_ (%)	0.2 ± 1.3	3.1 ± 7.9*	3.2 ± 9.8	0.015
${\dot{\bf V}}{{\bf{O}}_{\bf{2}}}$				
Highest ${\dot{\rm V}}{{\rm{O}}_{\rm{2}}}$ (mL·kg^−1^·min^−1^)	38.5 ± 4.5	38.3 ± 4.9	41.7 ± 7.5^#^	0.009
% Attained ${\dot{\rm V}}{{\rm{O}}_{\rm{2}}}\max $	62.0 ± 8.9	61.7 ± 9.0	66.9 ± 12.7^#^	0.009
Average ${\dot{\rm V}}{{\rm{O}}_{\rm{2}}}$ (mL·kg^−1^·min^−1^)	25.7 ± 2.9	25.1 ± 2.8	28.0 ± 4.8*^#^	<0.001
% Attained ${\dot{\rm V}}{{\rm{O}}_{\rm{2}}}\max $	41.5 ± 7.2	40.5 ± 6.9	45.3 ± 9.4^#^	<0.001
Time below to ${\dot{\rm V}}{{\rm{O}}_{\rm{2}}}$ at VT_1_ (%)	90.2 ± 14.7	95.3 ± 11.3*	84.0 ± 20.7^#^	0.001
Time between to ${\dot{\rm V}}{{\rm{O}}_{\rm{2}}}$ at VT_1_–VT_2_ (%)	9.0 ± 13.0	4.1 ± 10.0	12.0 ± 14.1^#^	0.024
Time above to ${\dot{\rm V}}{{\rm{O}}_{\rm{2}}}$ at VT_2_ (%)	0.8 ± 3.8	0.6 ± 3.4	4.0 ± 11.8	0.010

**Notes**:

Heart rate, oxygen uptake and time (expressed as relative percentages) in each metabolic zone, defined as below VT_1_, between the VT_1_ and VT_2_, and above the VT_2_ during each phase of the exergame session (*n* = 39).

Data are presented as the means ± standard deviation.

bpm, beats per minute; HR, heart rate; HRmax, maximum heart rate; N/A, not applicable; ${\dot {\rm V}}{{\rm O}_{\rm{2}}}$, oxygen uptake; VT_1_, first ventilatory threshold; VT_2_, second ventilatory threshold.

* Significant difference from ‘warm-up.’

# Significant difference from ‘main phase.’

The ${\dot{\rm V}}{{\rm{O}}_{\rm{2}}}$ behaviour during the *Sports Athlete* protocol is also shown in Table 3. The highest ${\dot{\rm V}}{{\rm{O}}_{\rm{2}}}$ attained in the ‘cool-down’ of the *Sports Athlete* protocol of the exergame *Hollywood Workout* was 8.8 ± 15.2%, higher than those attained in the ‘main phase’ (*p* = 0.009, effect size = 0.54 [medium]). The highest percent of ${\dot{\rm V}}{{\rm{O}}_{\rm{2}}}\max $ attained in the ‘cool-down’ was 8.8 ± 15.2%, higher than those attained in the ‘main phase’ (*p* = 0.009, effect size = 0.47 [small]). The average ${\dot{\rm V}}{{\rm{O}}_{\rm{2}}}$ attained in the ‘cool-down’ was 9.7 ± 16.9% and 11.5 ± 11.1%, higher than those attained in the ‘warm-up’ (*p* = 0.005, effect size = 0.58 [medium]) and ‘main phase’ (*p* < 0.001, effect size = 0.74 [medium]), respectively. The average percent of ${\dot{\rm V}}{{\rm{O}}_{\rm{2}}}\max $ attained in the ‘cool-down’ was 11.5 ± 11.1%, higher than those attained in ‘main phase’ (*p* < 0.001, effect size = 0.36 [small]), respectively.

As compared with ${\dot{\rm V}}{{\rm{O}}_{\rm{2}}}$ corresponding to VT_1_ and VT_2_, in the ‘warm-up,’ the average time spent at a ${\dot{\rm V}}{{\rm{O}}_{\rm{2}}}$ below the VT_1_, between the VT_1_ and VT_2_, and above the VT_2_ was 271 ± 44 s, 27 ± 39 s, and 2 ± 11 s, respectively. In the ‘main phase,’ the average time spent below the VT_1_ ${\dot{\rm V}}{{\rm{O}}_{\rm{2}}}$ between the VT_1_ and VT_2_, and above the VT_2_ was 1,048 ± 125 s, 45 ± 109 s, and 6 ± 37 s, respectively. Finally, in the ‘cool-down,’ the mean time spent below the VT_1_ ${\dot{\rm V}}{{\rm{O}}_{\rm{2}}}$, between the VT_1_ and VT_2_, and above the VT_2_ was 294 ± 87 s, 42 ± 49 s, and 14 ± 43 s, respectively.

Overall, HR and ${\dot{\rm V}}{{\rm{O}}_{\rm{2}}}$ of participants in the exergame session remained on average 1,503 ± 292 s (86.1 ± 16.7% of the total time session) and 1,610 ± 215 s (92.2 ± 12.3% of the total time session), respectively, below their VT_1_ values (Table 3).

The highest HR and percentage of HRmax in the exergame session were higher (96.4 ± 29.7% for both) than in the joystick session (*p* < 0.001 for both, effect size = 6.21 and 6.19 [large], respectively). Additionally, the average HR and percentage of HRmax in the exergame session were higher (83.1 ± 39.5 for both) than in the joystick session (*p* < 0.0001, effect size = 5.18 and 6.37 [large], respectively). Regarding ${\dot{\rm V}}{{\rm{O}}_{\rm{2}}}$, the highest ${\dot{\rm V}}{{\rm{O}}_{\rm{2}}}$ and percentage of ${\dot{\rm V}}{{\rm{O}}_{\rm{2}}}\max $ in the exergame session were four times (401.4 ± 120.2% for both) higher than in the joystick session (*p* < 0.001, effect size = 6.64 and 6.34 [large], respectively). The average ${\dot{\rm V}}{{\rm{O}}_{\rm{2}}}$ and percentage of ${\dot{\rm V}}{{\rm{O}}_{\rm{2}}}\max $ in the exergame session were four times (401.7 ± 84.3% for both) higher than in the joystick session (*p* < 0.001, effect size = 9.26 and 6.48 [large], respectively). For all times of the joystick session (25 min), average HR (73 ± 9 bpm) and ${\dot{\rm V}}{{\rm{O}}_{\rm{2}}}$ (5.3 ± 1.4 mL.kg^−1^.min^−1^) were spent below the VT_1_ levels, corresponding to 35.2 ± 5.0% of HRmax and 8.5 ± 1.8% of ${\dot{\rm V}}{{\rm{O}}_{\rm{2}}}\max $ of the participants, respectively. The highest HR (88 ± 12 bpm) and ${\dot{\rm V}}{{\rm{O}}_{\rm{2}}}$ values (9.2 ± 3.2 mL.kg^−1^.min^−1^) attained in the joystick session corresponded to 46.2 ± 6.9% of HRmax and 14.7 ± 4.6% of ${\dot{\rm V}}{{\rm{O}}_{\rm{2}}}\max $ of the participants, respectively. All variables in the joystick session presented lower values to the exergame session (*p* < 0.001).

## Discussion

The main aims of the present study were to investigate the responses of HR and ${\dot{\rm V}}{{\rm{O}}_{\rm{2}}}$ in young men during an exergame session (*Hollywood Workout on* *XBOX 360 Kinect®*) and compare with HR and ${\dot{\rm V}}{{\rm{O}}_{\rm{2}}}$ measured during joystick session. We also aimed to compare HR and ${\dot{\rm V}}{{\rm{O}}_{\rm{2}}}$ during the exergame session in relation to VT_1_ and VT_2_. As expected, physiological response values during the joystick session were lower than those evaluated during the exergame session. In addition, we found that the average intensity of the *Sports Athlete* protocol was ‘light to moderate,’ as an appreciable percentage (∼86% [HR] and ∼92% [${\dot{\rm V}}{{\rm{O}}_{\rm{2}}}$]) of the exercise bout occurred at an intensity lower than HR and ${\dot{\rm V}}{{\rm{O}}_{\rm{2}}}$ in VT_1_ levels (Stangier et al., 2016). Confirming this finding, the average percentage HRmax and percentage ${\dot{\rm V}}{{\rm{O}}_{\rm{2}}}\max $ during the *Sports Athlete* protocol were 70.7% and 41.6%, respectively.

These values are similar to data from other studies with exergames (Tan et al., 2002; Unnithan, Houser & Fernhall, 2006; Jordan, Donne & Fletcher, 2011) and contrary to other (Neves et al., 2015). Jordan, Donne & Fletcher (2011) reported that 15 healthy men (age 29 ± 4 years, body mass 81 ± 12 kg, height 1.77 ± 0.05 m, body mass index 25.9 ± 3.8 kg.m^−2^, ${\dot{\rm V}}{{\rm{O}}_{\rm{2}}}{\rm{peak}}$ 44.8 ± 5.5 ml.kg^−1^.min^−1^) attained ∼66% and ∼72% of HRmax and ∼41% and ∼56% of ${\dot{\rm V}}{{\rm{O}}_{\rm{2}}}\max $ during *Wii®* boxing and an exergame controlled only with movements of the lower limbs through the *PlayStation*® 2, respectively. Tan et al. (2002) reported that 40 young individuals (21 men and 19 women, age 17.5 ± 0.7 years) attained an average HR of 137 bpm (70% of HRmax), average ${\dot{\rm V}}{{\rm{O}}_{\rm{2}}}$ of 24.6 mL.kg^−1^.min^−1^ (44% of ${\dot{\rm V}}{{\rm{O}}_{\rm{2}}}\max $) during the exergame *Dance Dance Revolution 3rd Mix^™^ Konami®*. Unnithan, Houser & Fernhall (2006) reported that children and adolescents (11–17 years) attained ∼65% of HRmax during the exergame *Dance Dance Revolution^™^ Konami®*. On the other hand, average ${\dot{\rm V}}{{\rm{O}}_{\rm{2}}}$ (∼35% of ${\dot{\rm V}}{{\rm{O}}_{\rm{2}}}{\rm{peak}}$) did not reach the minimum values for development and maintenance of cardiorespiratory fitness (>46% of ${\dot{\rm V}}{{\rm{O}}_{\rm{2}}}\max $) proposed by the *American College of Sports Medicine* (ACSM) (Garber et al., 2011), similar to the results of the present study. Mills et al. (2013) and Lau et al. (2015) found similar results to the present study regarding percentage of HRmax in children during a high-intensity exergame (*Kinect Sports–200 m Hurdles*) and the exergame *I-Dong running*, respectively; however, the percentage of ${\dot{\rm V}}{{\rm{O}}_{\rm{2}}}\max $ reported by Mills et al. (2013) and Lau et al. (2015) was, respectively, lower (40.3%) and higher (61.4%) than that found in the present study. In general, despite previous studies investigating different populations and exercises, our results are in line with the literature. However, it is important to emphasize that the presented data should be extrapolated with caution.

According to the ACSM criteria, the intensity of the *Sports Athlete* protocol of the exergame *Hollywood Workout* can be classified as moderate, since it elicited average HR values corresponding to 70.7% of HRmax, framed in the range of 64–76% of HRmax proposed by the ACSM (Garber et al., 2011). The moderate intensity of the *Sports Athlete* protocol can be justified by fact that the evaluated participants presented high cardiorespiratory fitness, demanding a lower HR and ${\dot{\rm V}}{{\rm{O}}_{\rm{2}}}$ for the same workload when compared to sedentary individuals (DeMaria et al., 1978). In addition, the ACSM suggests that exercise must have a minimum intensity corresponding to 55% of HRmax to evoke the benefits of aerobic training (Garber et al., 2011). Therefore, the *Sports Athlete* protocol of the exergame *Hollywood Workout* can be a useful tool for improving cardiorespiratory fitness as well as being an alternative tool to traditional exercise protocols. However, it should be noted that for this purpose, the weekly exercise volume of at least 150 min (which would correspond to five exercise sessions, considering that each *Sports Athlete* protocol lasts approximately 30 min) must be met. An important advantage of the *Sports Athlete* protocol compared to classic aerobic activities (e.g. running, cycling) is that this protocol has exercises for both lower and upper limbs, thus being a training protocol for the whole body, which can increase ${{\dot{\rm V}}_E}$, HR, and ${\dot{\rm V}}{{\rm{O}}_{\rm{2}}}$ values to exercise through a greater amount of muscle mass involved (Jensen-Urstad, Svedenhag & Sahlin, 1994).

When evaluating the protocol in phases, significant differences were found between average HR of the ‘warm-up’ and the ‘main phase.’ As desired, the intensity of the ‘warm-up’ period was lower than the intensity of the ‘main phase,’ which follows the recommendations for exercise prescription (Powers & Howley, 2017). This is a very important aspect, since the ‘warm-up’ may be related to the reduction of the probability of muscular injuries due to stretches or dislocations (Woods, Bishop & Jones, 2007) in addition to influencing subsequent physical (Fradkin, Zazryn & Smoliga, 2010) and muscular performance (Mascarin et al., 2015). On the other hand, a significant increase of the HR and ${\dot{\rm V}}{{\rm{O}}_{\rm{2}}}$ occurred at the end of the ‘cool-down.’ This is contrary to the recommendations for exercise prescription (Pescatello et al., 2014; Powers & Howley, 2017), since the ‘cool-down’ should provide a gradual decrease of the HR (Powers & Howley, 2017). A possible explanation for the ‘cool-down’ intensity remaining high may be related to the *Mountain Climbers* exercise present in the ‘cool-down’ period, since the participants arrived very close to the ‘maximum effort’ (reported by participants), reflecting the amount of repetitions performed both in this exercise as in the others that constituted the ‘cool-down.’

### Strengths and limitations of the study

To the best of our knowledge, this is the first study to evaluate the intensity of an exergame through the HR and ${\dot{\rm V}}{{\rm{O}}_{\rm{2}}}$ values at VT_1_ and VT_2_ obtained from GXT. Most studies evaluated the intensity of exergames through percentages of the HRmax recommended by ACSM. The *Sports Athlete* protocol consists of exercises that people usually perform in their traditional routine of training (e.g. jog, bicycle crunches, push-ups, jump rope…), thus our results can be extrapolated to exercise facilities/gyms.

On the other hand, the participants in the present study performed all sessions coupled to the mouthpiece of the metabolic system. The discomfort generated by the salivation and fatigue of the chewing muscles (reported by the participants) may have interfered in the performance of the participants, although the performance of participants was better than suggested by the exergame. Thus, studies evaluating the influence of using the mouthpiece or mask over performance in exergames are needed, besides the accomplishment of the *Sports Athlete* protocol of the exergame *Hollywood Workout* in a non-laboratory context. Indeed, the study population included in this study were physically active. Therefore, future studies with sedentary individuals are needing to confirm our finding and understand the transferability of this findings to sedentary populations. Furthermore, the use of this protocol in the long term may be important to confirm the hypothesis that it would improve musculoskeletal and cardiorespiratory fitness, as well as possible changes in body composition as a function of energy expenditure. Blood lactate assessment could contribute to information on metabolic stress and anaerobic pathway contributions, since many participants reported high peripheral muscle fatigue. Nevertheless, we believe that these limitations do not prevent the conclusions of the study to be drawn.

## Conclusions

From the present data, it appears that the exergames used in the present study can be classified as light to moderate physical exercise for the participants evaluated. Indeed, during the exergames trial, ${\dot{\rm V}}{{\rm{O}}_{\rm{2}}}$ and HR remained predominantly below the VT_1_ level previously assessed during the GXT. Confirming this finding, the average ${\dot{\rm V}}{{\rm{O}}_{\rm{2}}}$ and HR during the exergames were according to ACSM’s recommendations. Thus, exergames could be an interesting alternative to traditional forms of exercise as a tool for increasing physical fitness.

## Supplemental Information

10.7717/peerj.5574/supp-1 Supplemental Information 1 Raw data.

## Additional Information and Declarations

### Data Availability

The following information was supplied regarding data availability: The raw measurements are provided in the Supplementary File.
